# Timing Effect on Transient Evoked Otoacoustic Emission Referral Rates for Newborn Hearing Screening within and after 48 Hours of Birth

**DOI:** 10.1055/s-0045-1802580

**Published:** 2025-07-29

**Authors:** Chuan Cheepcharoenrat, Amaraporn Rerkasem

**Affiliations:** 1 Department of Otorhinolaryngology – Head and Neck Surgery, Medical Education Center, Chiang Rai Prachanukroh Hospital, Chiang Rai, Thailand; 2 Center for Infectious Diseases and Substance Use, Research Institute for Health Sciences, Chiang Mai University, Chiang Rai, Thailand

**Keywords:** hearing loss, neonatal screening, otoacoustic emissions

## Abstract

**Introduction:**

Newborn hearing screenings must be completed within 48 hours of birth.

**Objective:**

To determine the optimal timing for the first transient evoked otoacoustic emissions (TEOAEs) test by comparing the referral rates of infants tested between different time intervals.

**Methods:**

The present study was a retrospective cohort analysis of 2,713 newborns who underwent TEOAE tests between February 2021 and June 2022. The infants were categorized into groups according to the age at which they were tested: 12 to 24 hours, 25 to 36 hours, 37 to 48 hours, and > 48 hours. We compared referral rates across these groups.

**Results:**

The overall referral rate for infants tested within 48 hours from birth was 53%, significantly higher than the rate for those tested after 48 hours (46%, *p* = 0.001). Notably, the highest referral rates were observed in neonates aged 25 to 36 hours. However, no significant difference in referral rates was found for high-risk infants tested either before or after 48 hours.

**Conclusion:**

Transient evoked otoacoustic emissions can be used to screen newborns' hearing within 48 hours, but the high referral rate suggests that Thailand should add automated auditory brainstem response (AABR) to its guidelines for hearing evaluation if newborns leave the hospital before 48 hours.

## Introduction

The worldwide incidence of bilateral hearing impairment among newborns is of 1.5 to 3.3 per 1 thousand individuals. 1 2 3 4 Congenital and progressive hearing loss, including cases that develop shortly after birth due to complications like hypoxia or ototoxic therapies, can lead to delayed speech development, mental and behavioral issues, and reduced social and cognitive abilities as the child begins schooling. 5 6 Early identification of infant hearing impairment before six months of age, coupled with the provision of intervention services within an average of two months postdiagnosis, can significantly enhance cognitive development compared to later identification. 7 Newborn hearing screening utilizing the transient evoked otoacoustic emission (TEOAE) test has been implemented for nearly two decades in certain Thai cities. 1 8 9 10 The TEOAE test is an effective screening method as it is specific, inexpensive, non-invasive, and does not require specialized neonatal examination skill. 11 12

Since Thai newborns must be discharged within 48 hours after birth to minimize hospital costs, conducting hearing screening during this period is crucial. However, while hearing screening can be performed after hospital discharge, some parents may need to bring their infants for follow-up tests, leading to delayed initial screenings. 4 Furthermore, performing hearing screening within 48 hours after birth may lead to a higher rate of test failure, necessitating repeat screenings. This, in turn, increases the workload, costs, and stress for both examiners and mothers. 13 Several factors contribute to the increased failure rate within this timeframe: residual amniotic fluid in the ear canal, transient middle ear effusion commonly seen in newborns, and the presence of vernix caseosa. These temporary conditions can interfere with sound transmission and test accuracy. Our study aims to determine the optimal timing for administering the initial TEOAE hearing test to newborns within the first 48 hours after birth. The objective was to identify the age at which hearing screening for newborns can be conducted to achieve a referral rate comparable to that of newborns screened after 48 hours of birth. Secondly, we sought to investigate whether these trends differed for high-risk and healthy neonates. The study compared the referral rates of infants who underwent hearing screening between 12 and 24 hours, 25 and 36 hours, 37 and 48 hours, and more than 48 hours after birth. If this study establishes a window of opportunity for the hearing screening within the first 48 hours after birth that is equivalent to screening after 48 hours, conducting the test during that window can reduce the need for retesting and the workload of examiners without compromising the results.

## Methods

### Study Population

The present study was conducted at a tertiary hospital and involved a retrospective cohort analysis of newborns who were born or admitted as inpatients between February 2021 and June 2022. All neonates born during this period were included in the study. Exclusion criteria comprised bilateral atresia or stenosis of the external ear canal, parental refusal to permit hearing testing, referral to another hospital, absence of hearing testing upon admission, or decedent status. A newborn is considered high risk if there is an evaluated likelihood of developing hearing loss. The Joint Committee on Infant Hearing's 2007 recommendations define infants at risk for hearing loss as those requiring neonatal intensive care for more than five days, including any of the following: extracorporeal membrane oxygenation (ECMO), assisted ventilation, exposure to ototoxic medications (gentamicin and tobramycin) or loop diuretics (furosemide), or having hyperbilirubinemia necessitating exchange transfusion irrespective of the length of stay. 14 Data from both healthy and high-risk newborns has been gathered for this study. The study was approved by the internal Ethical Committee of Research in Human Subjects with the Ref. nr.CR 0033.102/EC 588, which adheres to stringent ethical standards for research involving human participants.

### Instrumentation

The TEOAE test in the present study was conducted utilizing the Sentiero model (PATH medical GmbH, Germering, Germany). The process entailed placing a tiny probe into the newborn's ear canal to stimulate sound and record the response with a delicate microphone. In the presence of healthy cochlea, the outer hair cells respond by emitting a sound that is detected by the microphone. A trained nurse performed the screening in silence for less than 10 minutes. The equipment underwent automatic calibration before commencing the evaluation, and upon completion, the screening initiated.

The TEOAE test assessed frequency bands at 1,000, 2,000, 3,000, and 4,000 Hz. A pass criterion was defined as a signal-to-noise ratio (SNR) of ≥ 6 dB in at least three out of the four frequency bands. These criteria were uniformly applied to all infants' data. The test took place in a quiet room within the maternity ward, with ambient noise levels maintained below 50 dB sound pressure level. In cases of initial referral, immediate retesting was conducted to reduce false positives caused by transient factors such as probe placement or environmental noise.

The results were then displayed as either “pass” or “refer.” Bilateral “pass” responses in newborns were regarded as indicators of normal hearing, while infants who responded with a unilateral or bilateral “refer” were forwarded to the second-level screening.

### Data Management and Analysis

Medical records of all infants were reviewed for the present study. The medical data, including hearing screening results, were extracted manually from electronic medical records by a trained research assistant using a standardized data collection form. The extracted data were then independently verified by a second researcher to ensure accuracy. This process was applied consistently across all patient records included in the study.

Data collected included general characteristics, hearing loss risk factors, timing, and results of the first hearing test. The newborns were categorized into 4 groups based on the age at which they underwent the initial TEOAE screening: 12 to 24 hours, 25 to 36 hours, 37 to 48 hours, and more than 48 hours after birth. The data were analyzed using the t-, Ranksum, and Chi-squared tests. A *p* -value of < 0.05 was considered statistically significant.

## Results

**Fig. 1:**
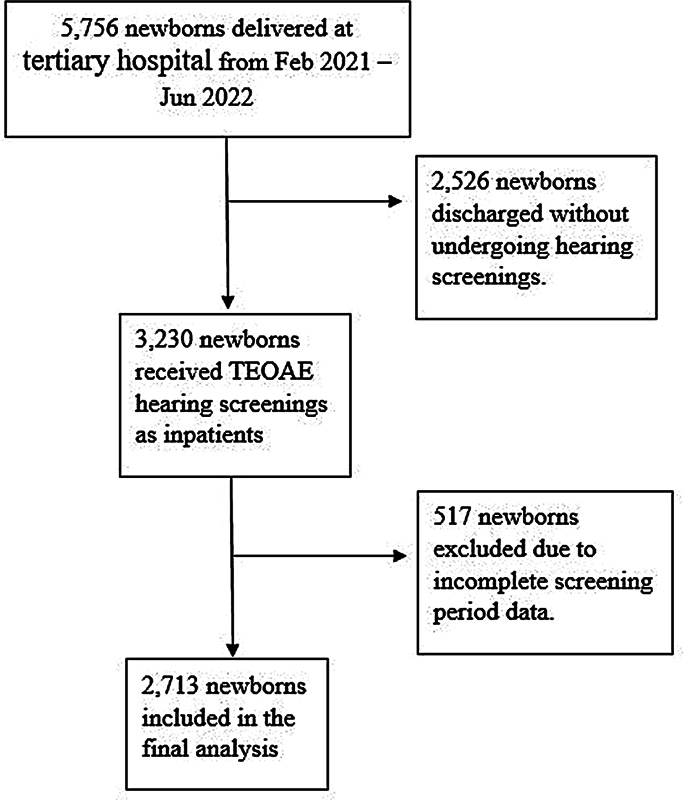
Flow diagram of newborn recruitment and transient evoked otoacoustic emission screening process.

During the study period from February 2021 to June 2022, a total of 5,756 newborns were delivered at the hospital. Among them, 3,230 underwent TEOAE tests prior to discharge, while 2,526 were discharged without testing but were scheduled for screening at the outpatient department. The records were incomplete for 517 of the 3,230 screened newborns, leaving 2,713 newborns for analysis ( Fig. 1 ).

The initial hearing screening for the newborns had an average age of 105.2 ± 222.3 hours, ranging from 15 to 3,264 hours, and a median age of 38 hours (interquartile range [IQR:] 29–70 hours). Approximately 67.9% of the newborns underwent hearing screening within 48 hours of birth, with 56.1% being screened between 25 and 48 hours. Only 12% underwent their first hearing screening after a week ( Table 1 ).

**Table 1:** Age distribution of newborns at the first transient evoked otoacoustic emission (TEOAE) screening

Age at first screening for TEOAE	N (%)
≤ 12 hours	0 (0.0)
13–24 hours (day 1)	321 (11.8)
25–48 hours (days 1–2)	1,522 (56.1)
49–72 hours (days 2–3)	257 (9.5)
73–96 hours (days 3–4)	115 (4.2)
97–120 hours (days 4–5)	81 (3.0)
121–144 hours (days 5–6)	55 (2.0)
145–168 hours (days 6–7)	38 (1.4)
167–720 hours (days 7–30)	259 (9.6)
721–1,440 hours (days 30–60)	51 (1.9)
> 1,441 hours (>60 days)	14 (0.5)
**Total**	2,713 (100)

The 2,713 newborns were categorized into four age groups according to the intervals at which hearing screening were conducted: 12 to 24 hours, more than 24 to 36 hours, more than 36 to 48 hours, and more than 48 hours ( Table 2 ). The majority of screenings were performed on infants who had been alive for 25 to 36 hours (976 infants) and those who had been alive for more than 48 hours (870 infants). Regarding gender, initial hearing screenings were conducted on two infants of unknown gender between the ages of more than 24 to 36 hours and more than 36 to 48 hours. Males underwent more screenings than females, particularly at ages above 48 hours. Newborns screened before 48 hours of age did not have an appearance, pulse, grimace, activity, and respiration (APGAR) score of 6, 5, and 3 cases at 1 minute, 5 minutes, and 10 minutes, respectively. There are significant differences in the distribution of APGAR scores at 1, 5, and 10 minutes, ( *p* < 0.001). All 144 (100%) newborns under 34 weeks gestation were screened for hearing after 48 hours. Significant differences were found in the categories of gestational age ( *p* < 0.001).

**Table 2:** Newborn characteristics on transient evoked otoacoustic emission screening across age intervals

Characteristics	N	Age at screening (hours)				*p* -value
		12–24	> 24–36	> 36–48	> 48	
Total number of screenings	2,713	321 (11.8)	976 (36.0)	546 (20.1)	870 (32.1)	N/A
Sex						
Male	1,464	158 (49.2)	471 (48.3)	286 (52.5)	549 (63.1)	< 0.001*
Female	1,247	163 (50.8)	504 (51.7)	259 (47.5)	321 (36.9)	
Ambiguous	2	0 (0)	1 (0.1)	1 (0.2)	0 (0)	
APGAR score at 1 minute						
0–3	45	0 (0)	0 (0)	0 (0)	45(5.2)	< 0.001*
4–5	51	1 (0.3)	5 (0.5)	3 (0.6)	42 (4.8)	
6–7	191	9 (2.8)	26 (2.7)	16 (2.9)	140 (16.1)	
8–10	2,412	311 (96.9)	943 (96.6)	523 (95.8)	635 (73.0)	
Missing	14	0 (0)	2 (0.2)	4 (0.7)	8 (0.9)	
APGAR score at 5 minutes						
0–3	10	0 (0)	0 (0)	0 (0)	10 (1.1)	< 0.001*
4–5	20	0 (0)	0 (0)	0 (0)	20 (2.3)	
6–7	66	1 (0.3)	4 (0.4)	2 (0.4)	59 (6.8)	
8–10	2,608	320 (99.7)	969 (99.3)	542 (99.2)	777 (89.3)	
Missing	9	0(0)	3 (0.3)	2 (0.4)	4 (0.5)	
APGAR score at 10 minutes						
0–3	2	0 (0)	0 (0)	0 (0)	2 (0.3)	< 0.001*
4–5	12	0 (0)	0 (0)	0 (0)	12 (1.4)	
6–7	37	0 (0)	1 (0.1)	1 (0.2)	35 (4.0)	
8–10	2,643	321 (100)	975 (99.9)	542 (99.3)	805 (92.5)	
Missing	19	0 (0)	0 (0)	3 (0.5)	16 (1.8)	
Gestational age (week, day)						
< 34 weeks	144	0 (0)	0 (0)	0 (0)	144(16.6)	< 0.001*
34–35 weeks, 6 days	133	6 (1.9)	9 (0.9)	10 (1.8)	108 (12.4)	
36–37 weeks, 6 days	509	55 (17.1)	172 (17.6)	108 (19.8)	174 (20.0)	
38–39 weeks, 6 days	1,484	198 (61.7)	608 (62.3)	352 (64.5)	326 (37.5)	
≥ 40 weeks	441	62 (19.3)	187 (19.2)	75 (13.7)	117 (13.4)	
Missing	2	0 (0)	0 (0)	1 (0.2)	1 (0.1)	
High-risk of hearing loss						
No	1,788	284 (88.5)	853 (87.4)	436 (79.8)	215 (24.7)	< 0.001*
Yes	917	37 (11.5)	117 (12.0)	109 (2.0)	654 (75.2)	
Missing	8	0 (0)	6 (0.6)	1 (0.2)	1 (0.1)	

Abbreviations: APGAR, appearance, pulse, grimace, activity, and respiration; N/A, not available.

**Notes:** The data represents the number (%) of infants in each characteristic subgroup screened at different age intervals. *Statistically significant.

A total of 917 infants, or 33.8% overall, were categorized as “yes” for the high risk of hearing loss, while 1,788 infants, or 65.9% overall, were classified as having “No” risk. The results indicate a statistically significant difference ( *p* < 0.001) between the screening age and all newborns. Interestingly, compared to infants without risk who are screened at a lower rate of 24.7%, infants at risk of hearing loss are screened more frequently (75.2%) at ages > 48 hours ( Table 2 ).

The overall referral rate for infants tested within 48 hours was 53%, significantly higher than the rate for those tested after 48 hours (46%, *p* = 0.001). However, referred rates for all newborns vary across different screening age intervals. The highest referred rate is observed in the screening age group between 25 and 36 hours (56.2%), while the lowest referred rate is found for screening age > 48 hours (46.2%). For high-risk newborns, referred rates do not show a significant difference among different screening age intervals. Referred rates for normal newborns also exhibit variation across different screening age intervals, with the highest referred rate observed in the screening age group between 25 and 36 hours (56.2%). There is a statistically significant difference among screening age groups ( *p* = 0.006) ( Table 3 ).

**Table 3:** Newborn referral rates on transient evoked otoacoustic emission hearing screening across different age intervals

Referral rate	Age at screening (hours)					*p* -value
	All age	12–24	> 24–36	> 36–48	> 48	
All newborns	1,381/2,713 (50.9)	164/321 (51.1)	548/976 (56.2)	267/546 (48.9)	402/870 (46.2)	< 0.001*
High-risk newborns	439/917 (47.9)	19/37 (51.3)	65/117 (55.6)	48/109 (44.0)	307/654 (46.9)	0.282
Normal newborns	937/1,788 (52.4)	145/284 (51.0)	479/853 (56.2)	219/436 (50.2)	94/215 (43.7)	0.006*

**Notes:** The data displays the number of infants referred to transient evoked otoacoustic emission /total infants screened (%) within each subgroup. *Statistically significant.

Table 4 presents TEOAE tests results, highlighting variations in referral rates based on the age at which newborns were screened. For overall newborns, referral rates for those screened within 12 to 24 hours and within 37 to 48 hours showed no significant difference compared to those screened after 48 hours ( *p* = 0.134 and *p* = 0.323 respectively). However, there was a statistically significant difference in referral rates between newborns aged between 25 and 36 hours and those screened after 48 hours ( *p* < 0.001).

**Table 4:** Comparison of referral rates for newborn transient evoked otoacoustic emission screening among different age groups relative to screening after 48 hours

Referral rate	Age at screening (hours)								
	12–24	> 48	*p* - *value*	> 24–36	> 48	*p* - *value*	> 36–48	> 48	*p* - *value*
All newborns	164/321 (51.1)	402/870 (46.2)	0.134	548/976 (56.2)	402/870 (46.2)	< 0.001*	267/546 (48.9)	402/870 (46.2)	0.323
High- risk newborns	19/37 (51.4)	307/654 (46.9)	0.601	65/117 (55.6)	307/654 (46.9)	0.086	48/109 (44.0)	307/654 (46.9)	0.573
Normal newborns	145/284 (51.1)	94/215 (43.7)	0.104	479/853 (56.2)	94/215 (43.7)	0.001*	219/436 (50.2)	94/215 (43.7)	0.118

**Notes:** The data displays the number of infants referred to transient evoked otoacoustic emission /total infants screened (%) within each subgroup. *Statistically significant.

Among the 4 age groups, the referral rate was notably higher for ages between 25 and 36 hours. Nevertheless, among high-risk hearing loss infants, no significant difference in referral rates was observed between those aged between 25 and 36 hours and those screened after 48 hours ( *p* = 0.086). In contrast, a significant difference in referral rates was found between normal newborns aged between 25 and 36 hours and those screened after 48 hours ( *p* = 0.001). Finally, no difference in referral rates was found between newborns screened between 37 and 48 hours and those screened after 48 hours, both in normal and high-risk groups ( *p* = 0.118 and 0.573 respectively) ( Table 4 ).

## Discussion

The study identified significant variations in referral rates among different age groups of newborns. This research aimed to determine the optimal time for newborn hearing screening within the first 48 hours. A primary objective was to enhance healthcare practices by evaluating referral rates and differences between high-risk and healthy neonates. Although overall referral rates were higher for infants tested within 48 hours, high-risk infants showed no significant differences across age categories. This indicates hearing screening is beneficial for high-risk populations regardless of timing. No significant differences were found between newborns screened within 12 to 24 hours or beyond 36 to 48 hours compared to those over 48 hours. A significant difference was observed between healthy newborns aged 25 to 36 hours and those over 48 hours. Thus, determining the optimal timing for hearing screening is complex due to varying referral rates.

Using data from 2,713 newborn screenings, this study evaluated screening effectiveness at various intervals before and after 48 hours. The diverse population included 1,788 healthy newborns and 917 high-risk infants. This study, extending beyond previous research, 8 9 aligns with Thailand's healthcare policy emphasizing shorter hospital stays and reduced costs. It highlights the flexibility of hearing tests for high-risk infants during the first 12 to 48 hours, adding valuable insights to neonatal care practices.

Our results revealed a significantly higher referral rate in the group screened in less than 48 hours compared to the average of 4% reported in previous research. 15 16 17 The presence of amniotic fluid remnants in the newborns' middle ears could explain the higher than average referral rate due to false positives during early screening. 18 19 Referral rates can be influenced by probe size, software options, external ear condition during screening, and baby management. 16 A prior study found that delaying the first TEOAE in neonates until they are at least 48 hours old reduces the referral rate. 17 20 21 The irregularities in referral rates after 48 hours warrant examining potential reasons, such as differences in screening procedures, demographic traits, or the specific healthcare setting of our study. 22 Additionally, variations in examiner proficiency, equipment calibration, or the frequency of specific risk factors could account for the higher refer rate. Further investigation of these variables is necessary to understand this incongruity. The screening results for newborns under 48 hours and over 48 hours varied, consistent with findings from other studies, 17 showing recurring trends in the newborn hearing test outcomes. More research is necessary to understand these variations and their implications.

Referral rates for healthy neonates between 25 and 36 hours and after 48 hours differed, while rates for those between 12 and 24 hours and 37 and 48 hours did not. Several factors can contribute to this. Hearing screening is affected by noise in the examination room, the mother's prenatal medication exposure, and examiner expertise. 23 Before discharged within 48 hours, newborns undergo various assessments, including blood tests and oxygen saturation tests. Most screening occurs between 25 and 36 hours after birth. Hearing screeners, who are nurses working in the ward, often perform multiple screening tasks simultaneously. This can lead to infants having trouble falling asleep, becoming restless, crying, or making noise disturbances during their own or other infants' examinations.

The absence of a statistically significant variation in the referral rates of high-risk infants can be attributed to several factors. Primarily, the shared high-risk status of these groups suggests a common susceptibility to hearing-related issues. Since these infants are already at an increased risk of developing hearing impairments due to various risk factors, screening within the first 48 hours may not significantly alter their referral rates. Overall, the lack of a notable variation in referral rates among high-risk infant groups underscores the importance of examining risk factors in influencing neonatal hearing outcomes.

The referral rates in this study for newborn hearing screening before and after 48 hours are comparable to those in other studies. 16 24 25 As newborns age, referral rates decrease, likely due to the resolution of middle ear fluid and debris within the first few days of life. The group with the most significant referral rate difference were healthy newborns aged 25 to 36 hours. Infants screened within 48 hours from birth are more likely to fail the initial test and require retesting, increasing the staff workload, reexamination cost, and mother's anxiety. While published referral rates ranged from 1.3 to 39%, 24 this study's single TEOAE test referral rate was as high as 50.9%. The results of neonatal hearing screening are influenced by the ambient noise level and the technical competence of the TEOAE.

Assessing the hearing of babies in Thailand within the first 48 hours from birth is highly challenging. To address the high referral rates found in this study, strategies need to be developed. It is recommended that the first TEOAE newborn hearing screening test be conducted on infants older than 48 to 72 hours to lower the referral rate. For neonates under 24 hours old with fluid-filled ears, the referral rate can reach up to 20%. 24 However, if the assessment is performed after 24 hours, the referral rate drops to less than 5%. 26 All babies undergo a TEOAE assessment, and those who fail receive a second TEOAE assessment. Infants who do not pass the second evaluation are diagnosed with an Automated Auditory Brainstem Response (AABR) test. The final decision and diagnosis are based on traditional auditory brainstem response (ABR) results, and infants with hearing loss received hearing and speech therapy.

The conclusions of the present study should be interpreted with caution due to inherent limitations. Firstly, out of 3,230 screened newborns, 517 had incomplete data. Although unfortunate, this necessitates careful interpretation of the overall results, as biases could have been introduced. However, the remaining 2,713 newborns with complete data still represented a large and diverse sample, making substantial bias unlikely. Secondly, the results cannot be broadly applied due to the single-center design in Thailand. The specific healthcare setting and local context may not reflect larger populations or various clinical settings. While this limits the generalizability of the results, the study site was a large tertiary care hospital with a high volume of births, providing relevant insights for similar hospital contexts within Thailand.

Another potential limitation was the 16-month study period, which could have been affected by unaccounted for changes in screening procedures, equipment calibration, or personnel. However, the hospital follows standardized universal newborn hearing screening protocols that are routinely audited, reducing the likelihood of systematic variations during the study timeframe. Additionally, while the risk stratification criteria adhered to accepted clinical guidelines, they may not have captured all potential risk factors for hearing impairment. A more detailed risk classification system could offer deeper insights into optimal screening timelines for various risk subgroups. Finally, the study focused solely on initial screening outcomes and referral rates, lacking long-term audiology follow-up data to confirm true versus false positive. Future studies with follow-up diagnostic assessments are necessary to validate screening performance across different age intervals.

Additionally, a key limitation of the current study is the lack of detailed frequency-specific data for referred tests. This information could have provided insights into which frequency bands were most affected, valuable for understanding the nature of early referrals and refining screening protocols. The equipment used in the study, designed for clinical screening rather than detailed research analysis, limited our ability to gather this data. Future studies could address this by using more advanced TEOAE equipment capable of providing frequency-specific data, or by incorporating additional diagnostic tests such as AABR for more comprehensive audiological profiles.

Despite these limitations, the current study provides important considerations for refining newborn hearing screening practices and guidelines tailored to the Thai healthcare context. The goal was to optimize the timing of the first screening step, making these first-line screening performance metrics directly relevant. The large sample size, standardized screening protocols, and well-defined objectives still offer valuable evidence to guide timing recommendations for newborn hearing screening within the first 48 hours of life. Further multi-center studies with comprehensive follow-up are warranted to validate and expand these findings.

Congenital hearing loss is not immediately noticeable at birth. Most children with hereditary hearing loss are born to parents with normal hearing and no health conditions or risk factors for hearing loss. 27 Failure to detect hearing loss in newborns early can negatively impact children's academic progress, growth, and language and speech development. 14 More efficient hearing screening needs to be promoted in Thailand. Although congenital hypothyroidism and phenylketonuria (PKU) are less common than hereditary hearing loss, neonatal screening for these disorders has advanced. In Thailand, the prevalence of PKU and congenital hypothyroidism is 0.3 per 100 thousand 28 and 1.69 per 10 thousand live births, 29 respectively. Combining TEOAE screening with AABR is an approach to reduce referral rates for neonatal hearing screening. 30 31 Although AABR is not yet included in the neonatal screening program at the study hospital, it is less affected by transient conductive pathology than TEOAE screening. Automated Auditory Brainstem Response screening is more costly as it takes longer and uses more resources. 20 However, it produces fewer false positives, has a lower referral rate, and results in fewer infants being lost to follow-up. Therefore, AABR is considered superior to TEOAE for universal newborn hearing screening. 31 32 33 Thailand should revise its guidelines to include additional tools like AABR to evaluate hearing if the newborn must leave the hospital before 48 hours.

## Conclusion

The findings underscore the complexities of implementing universal newborn hearing screening within the 48-hour postpartum window commonly practiced in Thailand. Screening newborns for hearing loss within 48 hours after birth is feasible, but the timing significantly impacts outcomes. According to the results of the present study, the optimal periods for conducting the initial TEOAE screening are either 12 to 24 hours or 37 to 48 hours postdelivery, as these intervals yielded referral rates comparable to those screened beyond 48 hours. However, the 24-to-36-hour window should likely be avoided due to elevated referral rates observed during this period. Further research is needed to optimize screening protocols for Thailand, particularly for high-risk infants who might benefit from earlier screening within the 48-hour window.
